# Intraoperative MRI-assisted neuro-port surgery for the resection of cerebral intraparenchymal cavernous malformation

**DOI:** 10.1186/s41016-019-0171-x

**Published:** 2019-09-11

**Authors:** Min Zhao, Changyu Lu, Jianfeng Liang, Yuanli Zhao, Xiaolei Chen

**Affiliations:** 1 Department of Neurosurgery, Peking University International Hospital, Beijing, China; 2 Department of Neurosurgery, Chinese PLA General Hospital, Beijing, China

**Keywords:** Intraoperative magnetic resonance image, Cavernous malformation, iMRI, Neuro-port surgery, Neuronavigation

## Abstract

**Background:**

Intraparenchymal cerebral cavernous malformation is difficult to localize intraoperatively with conventional frameless navigation due to the “brain shift” effect. We conducted this study to evaluate the efficacy and safety of intraoperative magnetic resonance image (iMRI)-assisted neuro-port surgery for the resection of cerebral intraparenchymal cavernous malformation.

**Methods:**

Between April 2016 and December 2017, 54 consecutive patients with intraparenchymal cerebral cavernous malformation who get surgical treatment in our hospital were enrolled into this study. Twenty-one patients were treated using iMRI-assisted neuro-port surgery (experiment group), and 33 patients underwent treatment by conventional microsurgery (control group). The iMRI was used in all cases for the compensation of the “brain shift” effect and keeping the navigation system up-to-date. The surgical resection rate, the total operation time, and the preoperative and postoperative Karnofsky Performance Status (KPS) scores were determined to evaluate the operative procedures.

**Results:**

There were no significant differences between the two groups in mean age, gender ratio, and volume of lesions (*P* > 0.05). For the experiment group, the average duration of the procedure was 188.8 min with total resection of the lesions achieved in all 21 cases. For the control group, the average duration of the procedure was 238.2 min with total resection of the lesions achieved in 25 of 33 cases. The differences in the average duration of the procedure and the number of totally resected lesions between the two groups were statistically significant (*P* < 0.05). Regarding postoperative neurological function, postoperative KPS scores for the experiment group were significantly higher than those of the control group (*P* = 0.018).

**Conclusion:**

Our results show that iMRI-assisted neuro-port surgery is helpful for intraparenchymal cerebral cavernous malformation surgery. The method provides high accuracy and efficiency for lesion targeting and permits excellent anatomic orientation. With the assistance of iMRI technology, we achieved a higher resection rate and a lower incidence of postoperative neurological deficits. Additionally, iMRI is helpful for the compensation of the “brain shift” effect, and it can update the navigation system.

**Electronic supplementary material:**

The online version of this article (10.1186/s41016-019-0171-x) contains supplementary material, which is available to authorized users.

## Background

Cavernous malformations (CMs) of the brain are vascular malformations with an estimated prevalence between 0.4 and 0.9% [1], appearing mainly as singular supratentorial lesions [2]. These lesions are made up of clusters of deformed vessels, lined by endothelium, and filled with blood at various stages of thrombosis. Most of these lesions are in the supratentorial area and are deep-seated in the brain.

The majority of patients do not need treatment, and radiosurgery is usually not recommended for the treatment of CMs. Lesions which lead to repeated bleeding and/or progressive neurological decline or intractable epilepsy do need resection [3, 4]. The CM-induced epilepsy originates in the glial scar layer and hemosiderin layer around the lesion. CM is one of the important causes of refractory epilepsy. The surgery should pursue the total resection of the CMs, the glial scar layer, and the hemosiderin layer around the lesion in order to eliminate or alleviate the symptoms of the patient, further improve the symptoms, and further improve the quality of life [5, 6].

However, surgery may be dangerous in patients with intracerebral deep-seated small CMs. The brain shift phenomenon after cerebrospinal fluid withdrawal and lesion removal has been a significant obstacle for surgeons. Brain tissue shifts during surgery can cause inaccurate localization during intraoperative neuronavigation. The use of a brain retractor could worsen the influence of the brain shift, and the continuous stretching may influence the blood supply of the cortex [7]. The risk of neurological damage is greatly increased when neurosurgeons attempt to remove more brain tissue to find the lesion. The development and utilization of intraoperative resection control imaging techniques, especially magnetic resonance imaging (MRI), can facilitate the localization and margin determination during intraaxial and extraaxial lesion resections [8, 9].

A greater awareness of the effect of maximal resection of brain lesions over the last decade has led to increased utilization of MRI as an intraoperative imaging technique for guidance in neurosurgical procedures [10, 11]. It is reported that intraoperative MRI (iMRI) and functional neuronavigation may be helpful to achieve successful surgical treatment [12–14]. Furthermore, diffusion tensor imaging has been applied in the preoperative and intraoperative management of these lesions. Combined use of iMRI with other neurosurgical modalities has also been attempted. Minimally invasive port surgery procedures have been reported for the microsurgery of intracranial small lesions [15] and for the treatment of supratentorial hypertensive intracerebral hemorrhage (HICH) [16]. With the development of iMRI and neuronavigation, neuro-port surgery is proving to be effective and may have some advantages compared with craniotomy. We present our initial experience with the iMRI-assisted neuro-port technique to treat cerebral intraparenchymal CMs, focusing on the outcome of this surgical approach.

## Methods

### General data

From April 2016 to December 2017, 54 consecutive CM patients who get surgical treatment in our hospital were enrolled in this study. They were confirmed to have CMs by postoperative pathological examination in our center. No patient died during the operations and the preoperative period. The experiment group consisted of 21 patients treated by iMRI-assisted neuro-port surgery. The control group consisted of 33 patients treated by microsurgery under the guidance of conventional neuronavigation. The research scheme was permitted by the Biomedical Ethics Committee. All patients and appointed agents signed the operation agreements and related documents.

### Clinical management

The operative methods for all patients were decided by preoperative discussions, combined with the wishes of the patients and their families. Deputy chief doctors and other senior doctors carried out the operations. Before the operations, all patients were examined by MRI to make operative plans. All 33 cases in the control group underwent microsurgery under the guidance of conventional neuronavigation.

All patients were scanned by high-field-strength superconducting magnet MRI in the diagnosis chamber of the iMRI operating room 1 or 2 days before the operation. Functional imaging includes blood oxygenation level-dependent functional MRI (BOLD-fMRI) and diffusion tensor imaging (DTI) fiber bundle tracing. BOLD-fMRI was used for the functional imaging of the cortex, a.o., the motor area, language area, and visual cortex. Preoperative imaging data were imported into the navigation planning workstation.

### Traditional microsurgery combined with neuronavigation system

The neuronavigation techniques used in the control group were the following. After anesthesia, the head was fixed by a head frame, and the tracer was fixed to the side of the frame. After registration, the position and adjacent structures of the lesion were shown in 3D and verified. Then, the surgical trajectory and incision were designed according to the information displayed. After applying a tracheal cannula and general anesthesia, the lesion was resected by microsurgery under the guidance of conventional neuronavigation. During the surgery, a navigation probe should be used to explore the lesion and the structures around it.

### iMRI-assisted neuro-port surgery

For the experiment group, we used a surgery system (IMRIS, Canada) with a high-field-strength superconducting magnet MRI machine (Siemens, Germany). After performing tracheal intubation and general anesthesia, a linear skin incision (4 cm) was designed according to the surface projection of the lesion shown in the navigation system. Craniotomy was performed, and the small bone flap was 2 cm in diameter. After opening the dura and combining the neuro-port with the navigation register, the working port was inserted toward to the lesion, guided by navigation. We used the microscope to provide lighting and observe the lesion; then, we used an aspirator and bipolar electrocoagulation for lesion resection, hemostasis, etc. After resection of the lesion, iMRI was performed to observe the degree of lesion resection, and we accordingly decided whether it was necessary to update the navigation scheme and continue the resection. After successful resection and wound hemostasis, we withdrew the neuro-port, sutured the dura, replaced the bone flap, and closed the skull conventionally.

### Observation indices and postoperative follow-up

A postoperative MRI scan was performed 5 to 10 days after surgery for every patient. After postoperative MRI examination, the cases with DICOM image data were retained, and we calculated the tumor volume by 3DSlicer. We calculated the degree of surgical resection using the following formula: Surgical resection degree = (preoperative tumor volume − postoperative residual tumor volume)/preoperative tumor volume × 100%. Cases with a surgical resection degree ≥ 90% were considered to have achieved total tumor resection; cases with < 90% were considered to have achieved partial tumor resection. We evaluate the KPS score when patients leave the hospital. The surgical resection rate, total operative time, and postoperative KPS scores were recorded for all patients to evaluate the operative procedures.

### Statistical analysis

SPSS 19.0 was used to do the statistical analysis. The mean age, lesion volume, lesion resection rate, mean operation time, and KPS scores of the experiment group and the control group were compared by the independent sample *t* test. The difference in sex ratio was analyzed by chi-square test, and the threshold was set at α = 0.05.

## Results

Regarding the comparison of the general information in the two groups, there were no statistically significant differences between the two groups in mean age, gender, and lesion volume. The preoperative KPS scores between the two groups showed no statistically significant difference (*P* = 0.858).

Regarding the comparison of the intraoperative observation indices of the two groups, the mean operation time of the experiment group was shorter than that of the control group (*t* = 1.362, *P* = 0.029). Compared with microsurgery under the guidance of conventional neuronavigation, iMRI-assisted neuro-port surgery took less time (*t* = 8.623, *P* = 0.026). The postoperative KPS scores between the two groups showed no statistical significance (*P* = 0.8146). The total resection rate in the experiment group was significantly higher than that in the control group (*P* = 0.017) (Additional file 1).

**Fig. 1:**
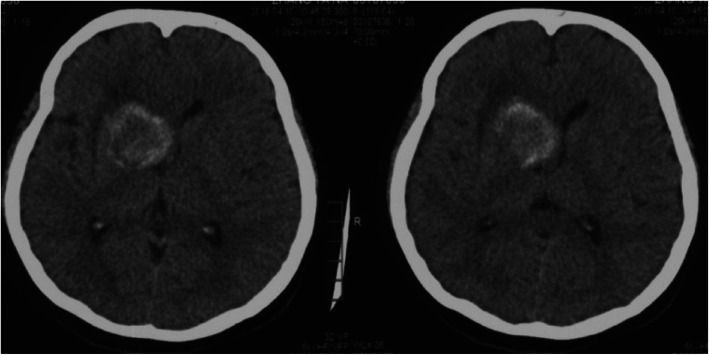
Preoperative CT scan. It showed an occupancy with hemorrhage

**Fig. 2:**
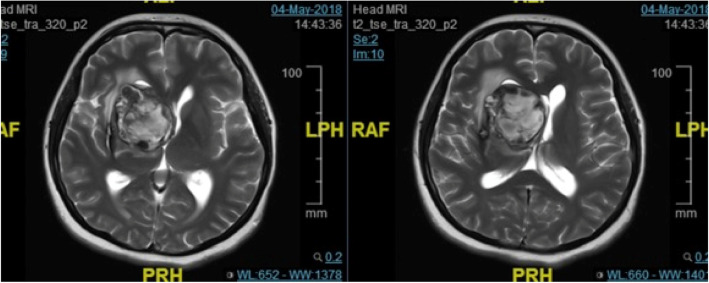
Preoperative T2flair inspection. It showed occupancy with hemorrhage more clearly

**Fig. 3:**
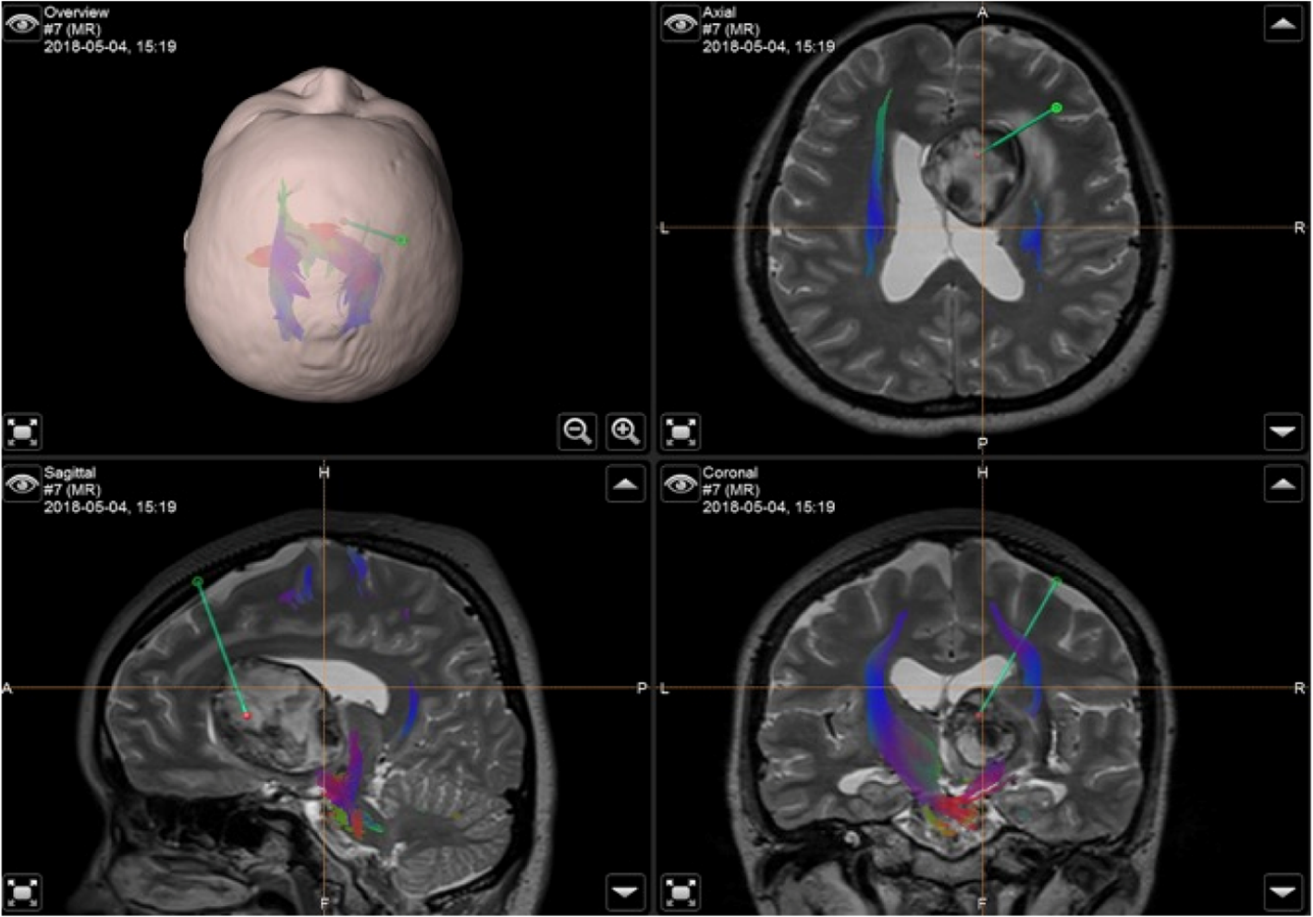
Operative navigation plan. It showed reconstruction of DTI under navigation and design of the surgical approach

**Fig. 4:**
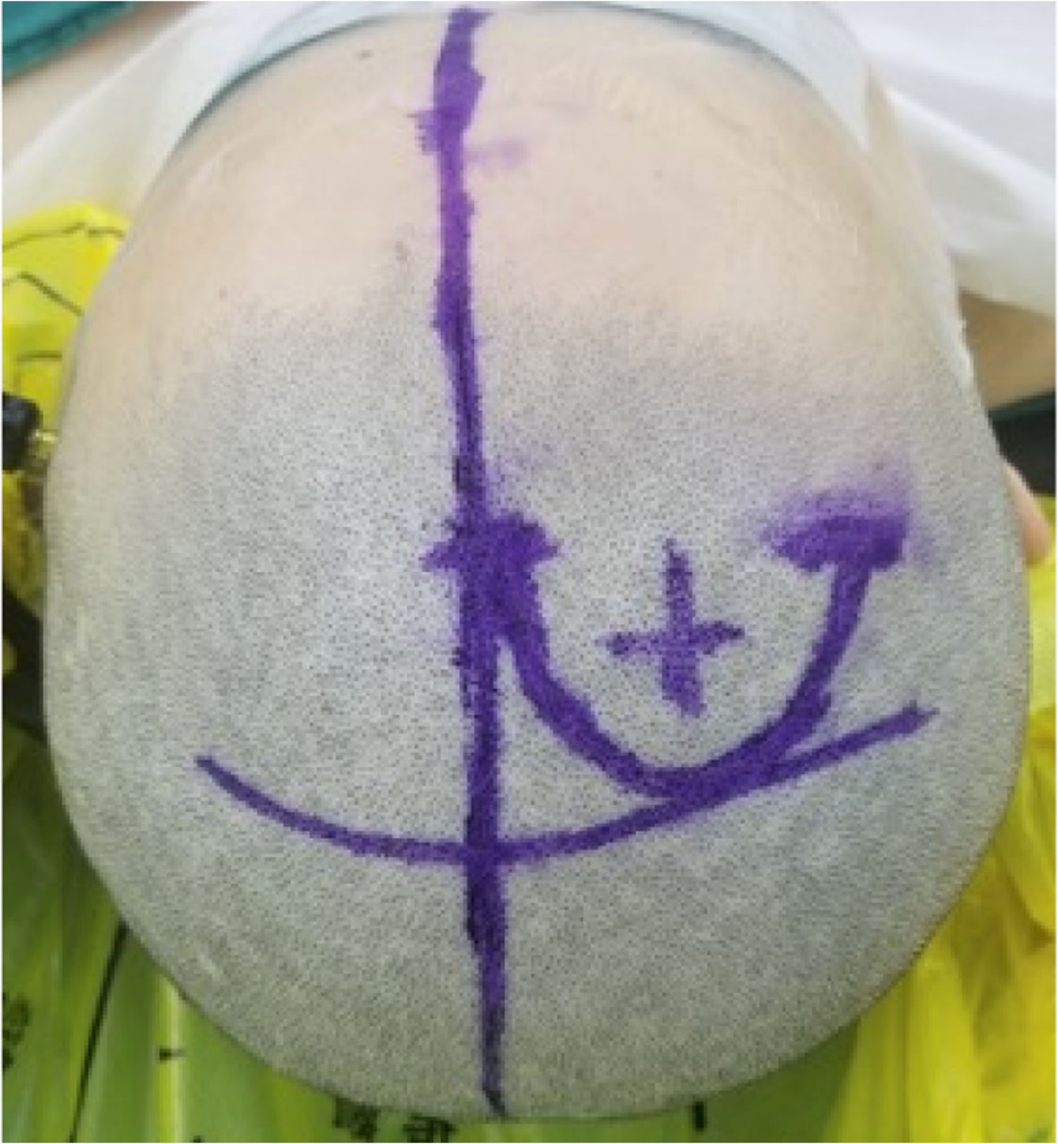
Operative incision. It showed an arc incision before coronal suture on the scalp

**Fig. 5:**
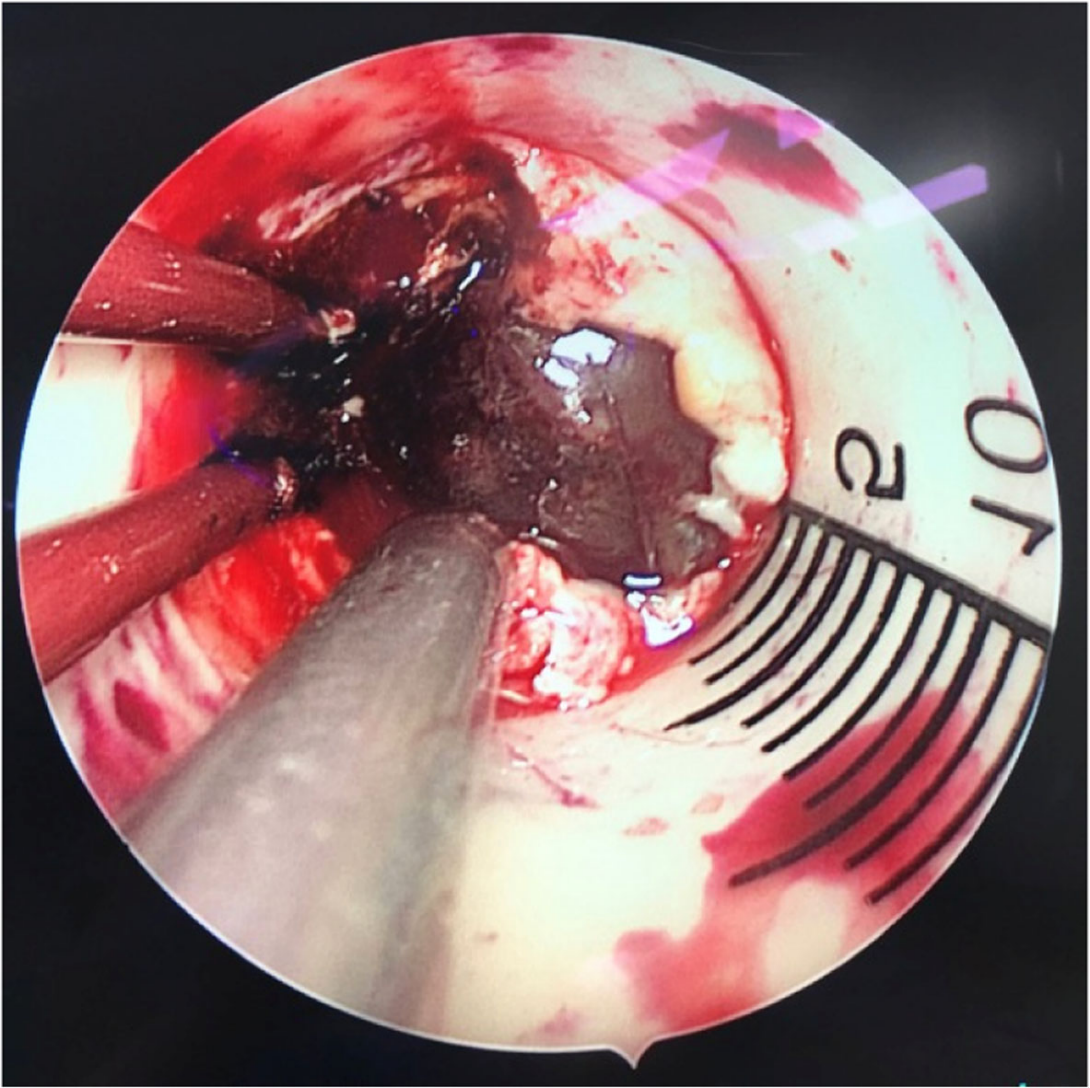
Operative field. It showed the vision of surgical microscope through the neuro-port canal

**Fig. 6:**
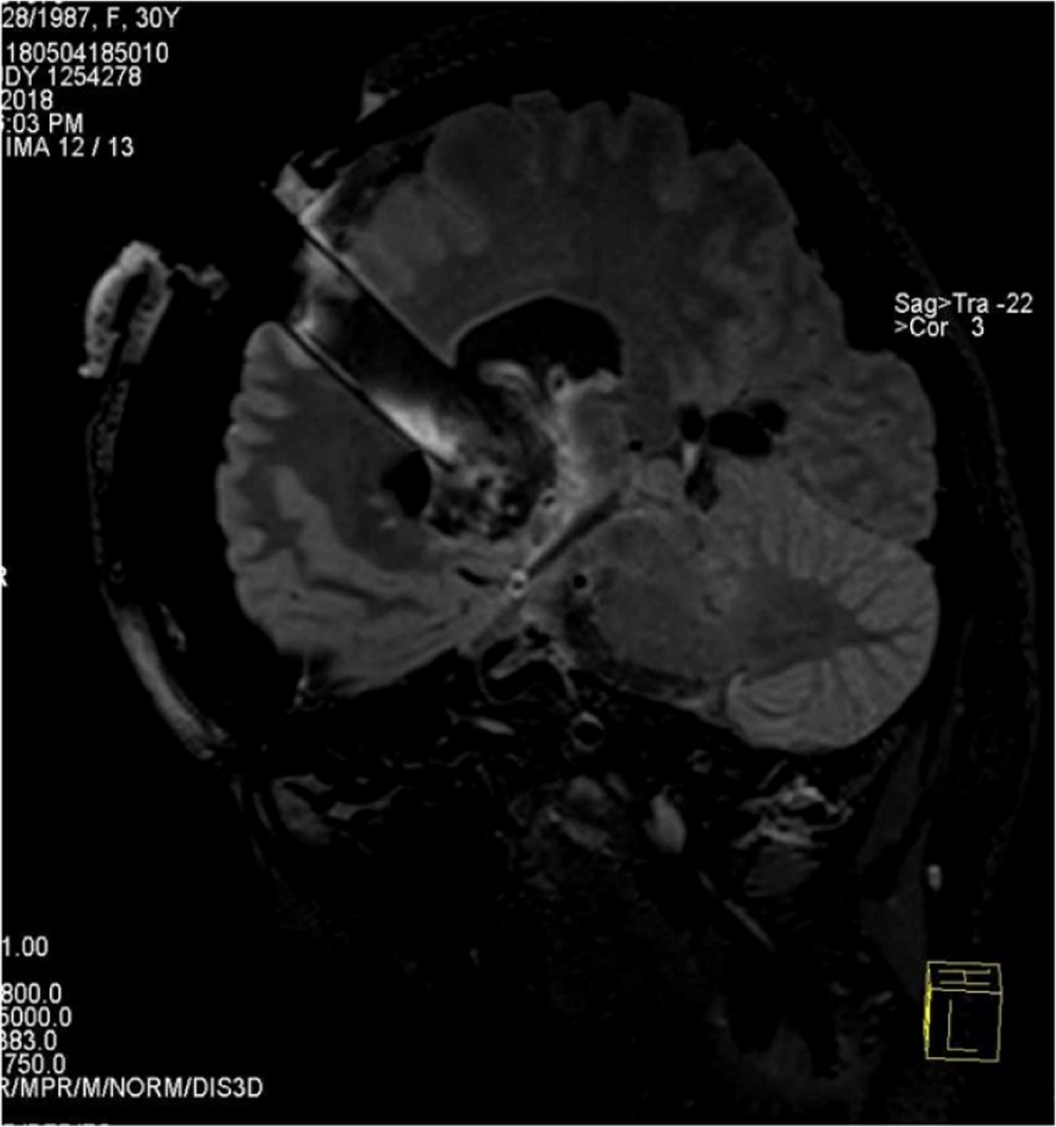
Intraoperative iMRI scan. T1 inspection showed that the occupancy and hemorrhage were totally removed

**Fig. 7:**
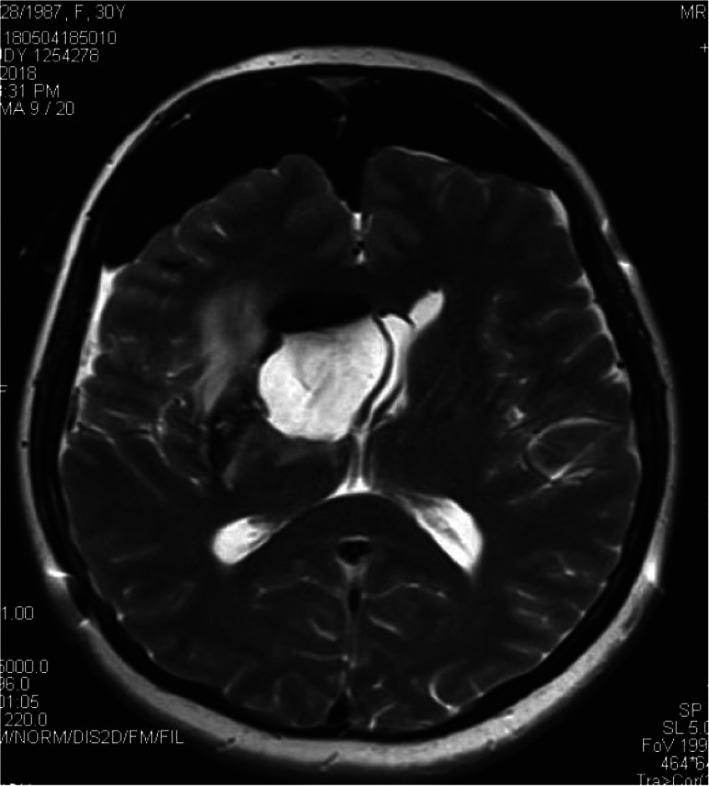
Intraoperative iMRI scan. T2 inspection showed that the occupancy and hemorrhage were totally removed

**Fig. 8:**
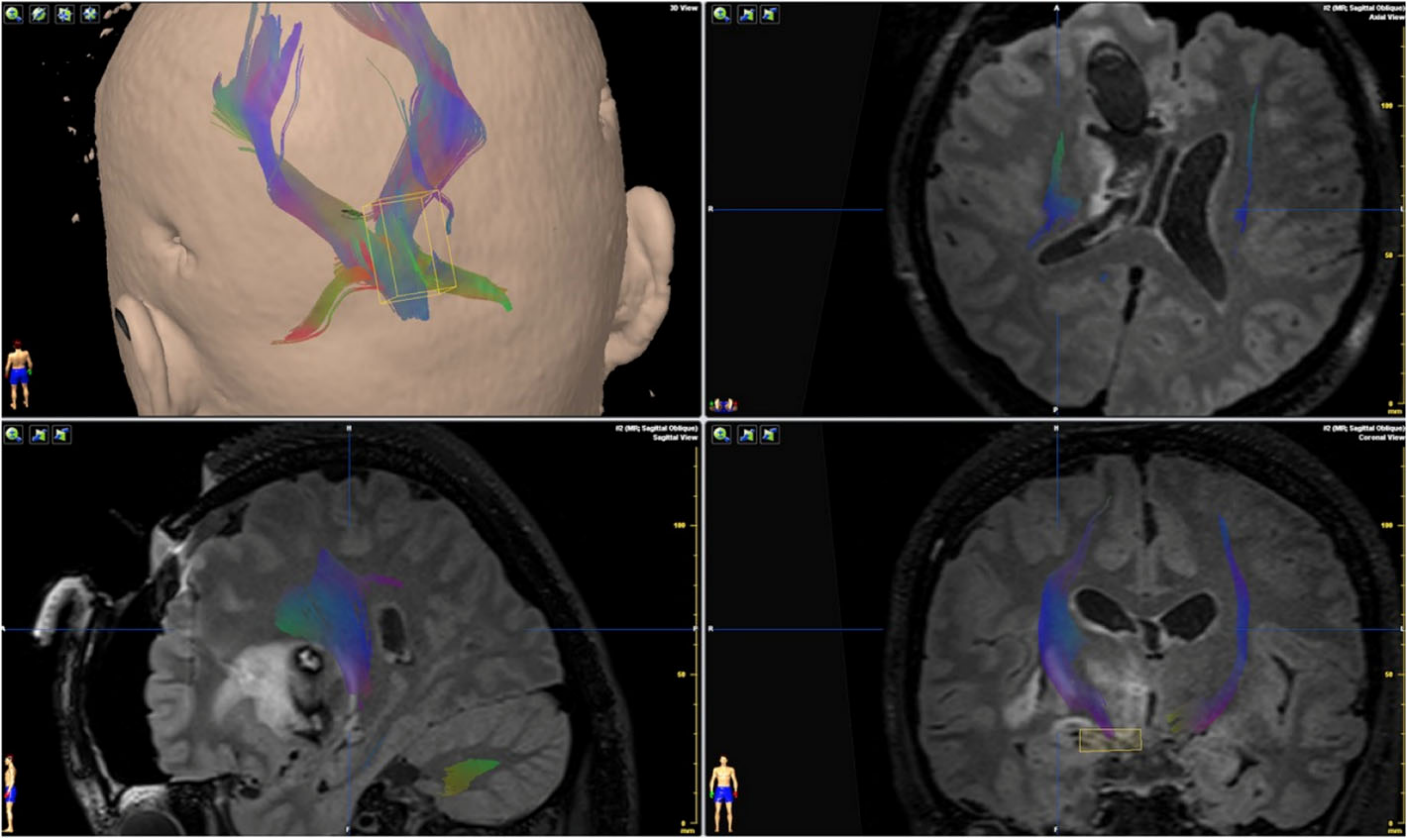
Postoperative DTI reconstruction. It showed that the fasciculus was well preserved after surgery

**Fig. 9:**
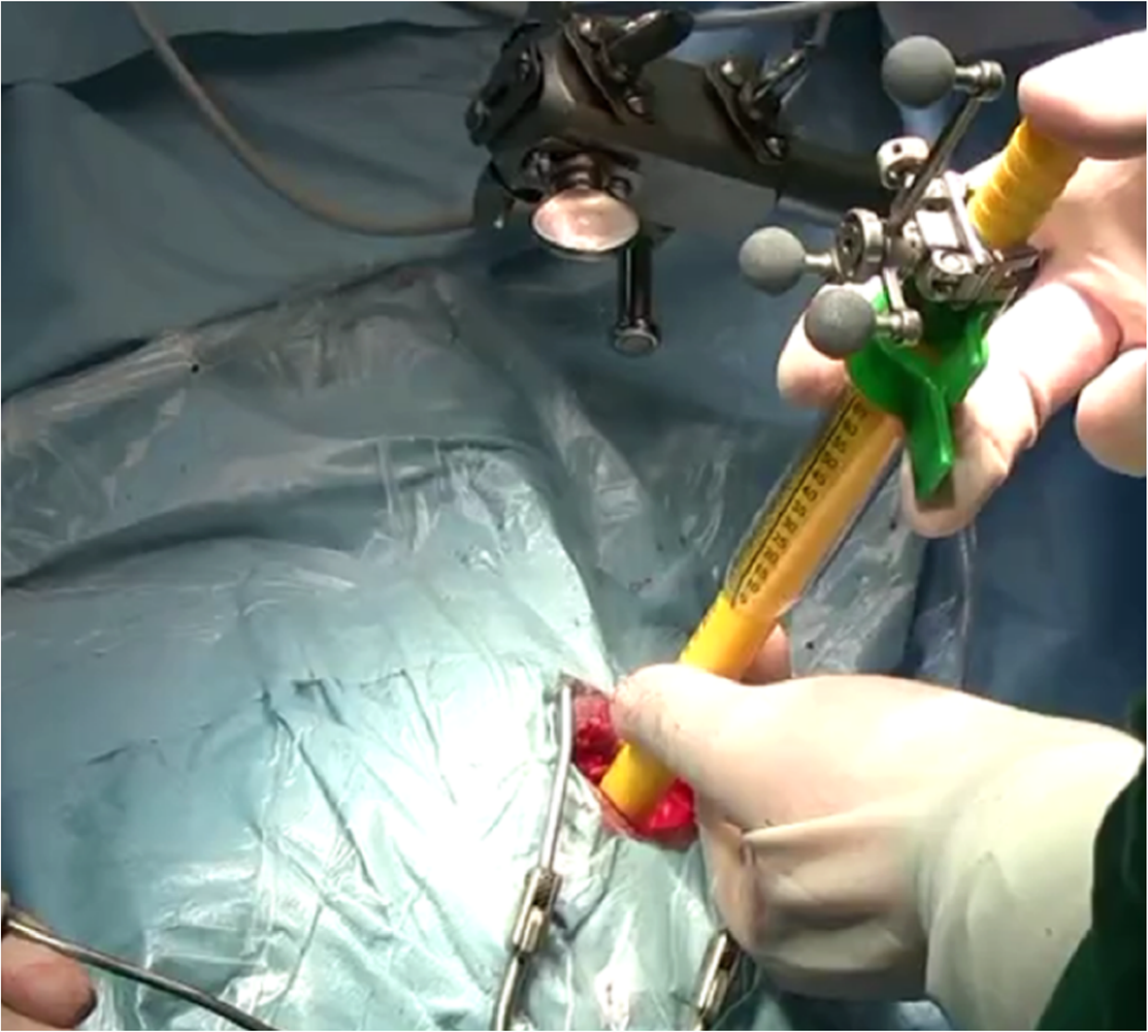
The neuro-port instrument. It showed the fixing and navigation registering part of the instrument preoperative

In order to illustrate the strength of the iMRI-assisted neuro-port surgery, we here describe a typical case. A 56-year-old male patient was admitted to the hospital due to sustained seizures for more than 2 months. Preoperative MRI suggested the presence of a deep-seated lesion in the left temporal lobe. The volume of the lesion was 2.7 cm^3^. The case was included in the experiment group; we used iMRI, neuro-port, and neuronavigation to resect the lesion completely. Postoperative pathology is CM. After the operation, the symptom of the patient’s epilepsy was relieved entirely. The lesions showed no recurrence during the 12-month follow-up (see Figs. 1, 2, 3, 4, 5, 6, 7, 8 and 9).

## Discussion

Neuronavigation is useful in designing operative trajectories, improving the resection degree of lesions, and reducing related complications. Conventional navigation technology relies on preoperative eikonic data. Consequences of gravity action, e.g., the loss of cerebrospinal fluid and brain retraction, could make brain shift inevitable and lead to a decrease in navigation accuracy. By analyzing the brain shift with 0.2-T magnetic resonance, Nimsky proved that it shows a strong variability; in his research, the distance of the cortex shift could reach 24 mm during surgery, and deep-seated lesions could shift more than 3 mm. The decrease in navigation accuracy could result in lesion localization failure during surgery.

The port retractor system was first introduced by Kelly et al. in 1988 for the excision of intraparenchymal lesions [17]. In our experiment group, we use navigation to guide neuro-port reaching the lesion at the beginning of surgery, do a small craniectomy and release fewer cerebrospinal fluid, and fixated the neuro-port instrument all the procedure, therefore we can reduce time of exposing the lesion and avoid brain shift. iMRI-assisted neuro-port surgery can avoid prolonged operation times by repeated intraoperative imaging. Imaging techniques such as fMRI and DTI are commonly used in coordination with standard neuronavigation MRI to preoperatively plan the entry point and the trajectory of the port. This planning is used to determine the appropriate incision and craniotomy location and size [15]. In port surgery, craniotomy with a small bone flap (bone window 2 cm) was performed, and the working port was sent to the anatomical position of the lesion, which was then observed and cut through the port. Some studies have demonstrated that the port redistributes pressure more equally in the surrounding tissue, and it causes less direct cutting and/or tearing trauma to the surrounding brain tissue [18, 19]. Also, patient morbidity can decrease thanks to the decrease in potential injury to the critical white matter tracts and vascular pedicles of both cortical and subcortical structures [20].

With recent improvements in microscope technology, the possibility of stereoscopic visualization through narrow spaces has become possible, opening opportunities for microscope-assisted port surgery [21]. Surgeons can make small adjustments in the trajectory of the port during surgery in order to visualize various parts of the target lesion.

The contrast in total operation time between the experiment group and the control group showed that iMRI-assisted neuro-port surgery could reduce the surgery time significantly. Using iMRI, we could visualize the tumor resection in real time and determine the spatial position of the tumor. During the operation, with the help of DTI and other special features of MRI, we could quickly reconstruct the white matter fiber bundles and other important nerve structures, update the operation plan, and reduce the technique-related complications that could cause functional neurological disorders. The iMRI-assisted neuro-port can provide important help to locate intracranial CMs rapidly and accurately. These techniques are feasible, safe, and minimally invasive.

## Conclusions

A minimally invasive neurosurgical approach using iMRI combined with a neuro-port can provide a safe and effective option for patients with cerebral intraparenchymal CMs. The method provides high accuracy and efficiency for lesion targeting and permits excellent anatomic orientation. Also, iMRI is helpful for the compensation of the “brain shift” effect and for the up-to-date navigation. Our findings indicate that neuro-port surgery may improve the prognosis and quality of life of patients with CMs.

It should be noted that our study has limitations. First, data were collected retrospectively, and there might have been selection bias. Second, our study used a relatively small sample size, which does not allow us to draw strong conclusions but rather generate hypotheses. The research still requires a larger sample of control studies to confirm the clinical value of the method.

## Additional file

Additional file 1: Preoperative and postoperative sample analysis. (DOCX 18 kb)

## Data Availability

The datasets used and/or analyzed during the current study are available from the corresponding author on reasonable request.

## Abbreviations

BOLD-fMRI: Blood oxygenation level-dependent functional MRI
CM: Cavernous malformation
DTI: Diffusion tensor imaging
HICH: Hypertensive intracerebral hemorrhage
iMRI: Intraoperative magnetic resonance image
KPS score: Karnofsky Performance Status score
