# Supplementary material for: Intraoperative MRI-assisted neuro-port surgery for the resection of cerebral intraparenchymal cavernous malformation
Source: Chin Neurosurg J. 2019 Sep 11;5:23. doi: 10.1186/s41016-019-0171-x (PMC7398321; doi:10.1186/s41016-019-0171-x)
Supplement: Supplementary file 1 — Preoperative and postoperative sample analysis. (DOCX 18 kb) [file 41016_2019_171_MOESM1_ESM.docx]

**Table: Preoperative and postoperative sample analysis.**

|  | Control group(n=21) | | Experimental group(n=33) | | | *P*-value |
| --- | --- | --- | --- | --- | --- | --- |
| Age(yrs) | | 34.9±11.0 | | 39.9±15.8 | 0.2151 | |
| Lesion volume(cm^3^) | | 5.5±3.1 | | 5.7±4.1 | 0.8888 | |
| female | | 28.6% (6/21) | | 39.4% (13/33) | 0.603 | |
| Operation time(min) | | 238.2±22.0 | | 188.8±16.6 | 0.000 | |
| Resection rate | | 75.8% (25/33) | | 100% (21/21) | 0.017 | |
| Preoperative KPS | | 90 (90-90) | | 90 (90-90) | 0.858 | |
| Postoperative KPS | | 90 (90-90) | | 90 (90-90) | 0.8146 | |

1. **Test of normality**

| group | variable | SW-value | *P*-value |
| --- | --- | --- | --- |
| Experimental group | Age | 0.966 | 0.654 |
| Control group | Age | 0.965 | 0.359 |
| Experimental group | Size | 0.931 | 0.146 |
| Control group | Size | 0.936 | 0.053 |
| Experimental group | Operation time | 0.977 | 0.884 |
| Control group | Operation time | 0.937 | 0.056 |

The age, size, and operation time in the control group and experimental group follow a normal distribution. The independent sample t-test is used for statistical analysis.

**2. Preoperative sample analysis**

(1) The independent sample t-test (age, lesion size)

| Index | Control group | | Experimental group | | t-value | *P*-value |
| --- | --- | --- | --- | --- | --- | --- |
|  | Mean value | Standard deviation | Mean value | Standard deviation |  |  |
| Age(yrs) | 34.87879 | 10.97941 | 39.85714 | 15.77113 | 1.2646 | 0.2151 |
| Size(cm^3^) | 5.503939 | 3.069897 | 5.650952 | 4.106606 | 0.1409 | 0.8888 |

In accordance with the results of the independent sample t-test, we concluded there is no statistically significant difference between the control group and the experimental group in age (*P* > 0.05) and in lesion size (*P* > 0.05).

(2) Chi-square test (sex)

| Group | female | male |  | Chi square value | *P*-value |
| --- | --- | --- | --- | --- | --- |
| Experimental group | 6 | 15 |  | 0.27 | 0.603 |
| Control group | 13 | 20 |  |  |  |

The chi-square test shows there is no statistically significant difference in sex ration between the experimental group and the control group (*P* > 0.05).

(3) Rank sum test (preoperative KPS)

| Index | Control group | | Experimental group | | W-value | P-value |
| --- | --- | --- | --- | --- | --- | --- |
|  | Median | Quartile range | Median | Quartile range |  |  |
| Preoperative KPS | 90 | 0 | 90 | 0 | 342 | 0.858 |

The Wilcoxon rank-sum test shows no statistically significant difference between the preoperative KPS values of the experimental group and the control group (*P* > 0.05).

**3. Postoperative sample analysis**

| (1) Independent-samples t-test (operation time) | | |  |  |  |  |
| --- | --- | --- | --- | --- | --- | --- |
| Index | Control group | | Experimental group | | t-value | *P*-value |
|  | Mean value | Standard deviation | Mean value | Standard deviation |  |  |
| Operation time(min) | 238.2121 | 22.00534 | 188.7619 | 16.57077 | -9.3873 | <0.01 |

Following the independent-samples t-test, the operation time of the experimental group was significantly shorter than that of the control group (*P* < 0.01).

| (2) Fisher’s test (resection rate) | | | | | |  |
| --- | --- | --- | --- | --- | --- | --- |
| Group | | | Total resection | | Partial resection | *P*-value |
| Experimental group | | 21 | | 0 | | 0.017 |
| Control group | 25 | | | 8 | |  |

The number of partial resections in the experimental group was zero, so for the comparison between the resection rates of the experimental group and the control group we used the Fisher test. The resection rate of the two groups was statistically different; the total resection rate in the experimental group was higher than that in the control group.

| (3) Rank-sum test (postoperative KPS) | | |  | |  | |  | | |  |  |
| --- | --- | --- | --- | --- | --- | --- | --- | --- | --- | --- | --- |
| Index | Control group | | Experimental group | | | W= | | P= |  |  |  |
|  | Median | Quartile range | Median | Quartile range | |  |  |  |  |  |  |
| Postoperative KPS | 90 | 0 | 90 | 0 | | 340 | | 0.8146 |  |  |  |

The Wilcoxon rank-sum test shows no statistically significant difference between the postoperative KPS values of the experimental group and the control group (*P* > 0.05).

**4. intraoperative date: cases of reoperation after iMRI reexamination in experiment group**

7 cases received reoperation after iMRI scan intraoperation in all 21 patients, the remaining 14 patients have total resection of the lesion by one operation before the scan.
